# Clinical Utility of ^18^F-PSMA-1007 Positron Emission Tomography/Magnetic Resonance Imaging in Prostate Cancer: A Single-Center Experience

**DOI:** 10.3389/fonc.2020.612701

**Published:** 2021-02-11

**Authors:** Ao Liu, Miao Zhang, Hai Huang, Chuanjie Zhang, Xiaohao Ruan, Wenhao Lin, Biao Li, Lu Chen, Danfeng Xu

**Affiliations:** ^1^ Department of Urinary Surgery, Ruijin Hospital, Shanghai Jiaotong University School of Medicine, Shanghai, China; ^2^ Department of Nuclear Medicine, Ruijin Hospital, Shanghai Jiaotong University School of Medicine, Shanghai, China

**Keywords:** prostate specific membrane antigen, positron emission tomography, magnetic resonance imaging, prostate cancer, management

## Abstract

**Purpose:**

This study aimed to evaluate the clinical utility of ^18^F-PSMA-1007 positron emission tomography (PSMA PET)/magnetic resonance imaging (MRI) imaging in patients with suspected or defined prostate cancer.

**Methods:**

In the pilot study, we retrospectively investigated 62 patients who underwent PSMA-PET/MRI for suspected or defined PCa between June 2019 and June 2020. Patients were grouped into three subgroups: (1) suspected PCa without histological evidence, (2) primary PCa, (3) biochemical recurrent prostate cancer (BRPCa). Two nuclear physicians independently interpreted the results of PSMA-PET/MRI. Management strategies before PSMA-PET/MRI were retrospectively reported, and the management strategy was re-evaluated for each patient considering the PSMA-PET/MRI result. The changes in strategies were recorded. Besides, the correlation between prostate specific antigen (PSA) level and management changes was also accessed by Fisher exact test, and two-side p < 0.05 was assumed as statistical significance.

**Results:**

There were 28 patients in the suspected PCa group (group 1), 12 in the primary PCa group (group 2), and 22 in the BRPCa group (group 3). Overall, the intended decisions were changed in 26 (41.9%) of 62 patients after PSMA-PET/MRI, including 11/28 (39.3%) in suspected PCa group, 1/12 (8.4%) in primary PCa group, and 14/24 (63.6%) in BCR group. In group 1, the main impact on subsequent management included decreased active surveillance (from 20 to 9) and increased prostate biopsy (from 8 to 19). PSA levels were not significantly associated with management changes in suspected PCa patients (p = 0.865). In group 2, the main impact on subsequent management included decreased radical surgery (from 8 to 7), and multimodal therapy appearance (n = 1). Only in the category of PSA levels of ≥20 ng/ml, the management of primary PCa was changed. In group 3, the main impact on subsequent management included decreased salvage radiotherapy (from 5 to 2), increased systemic therapy (from 6 to 7), and increased multimodal therapy (from 11 to 13). The highest proportion of management changes occurred in BCR patients with 0.5≤PSA<1 ng/ml.

**Conclusion:**

From our preliminary experience, PSMA-PET/MRI may be a valued tool for defining PCa lesions and changing management. The biggest impact of management intent was in patients with BRPCa, especially in patients with 0.5≤PSA<1 ng/ml. However, further studies are needed to confirm our pilot findings.

## Introduction

Prostate cancer (PCa) is the most frequent malignancy in men in the western world (1). In China, though lower incidence rate, significantly increased incidence and mortality of PCa are worth to rise our guard (2). Multi-parametric magnetic resonance imaging (mpMRI) is a standard imaging technique in the field of PCa, and confirmed its value in improving the detection of clinically significant PCa (csPCa) and guiding prostate biopsy (3). However, missed diagnoses of PCa and unnecessary biopsies are still unavoidable (4). For primary PCa, localized or locally advanced PCa is mainly treated with radical prostatectomy (RP), while metastatic PCa require systemic treatment *via* androgen deprivation therapy (ADT) or chemotherapy. Nevertheless, exact local and whole-body staging in a single investigation remains a challenge with conventional imaging techniques. Additionally, after primary treatment, increasing serum PSA levels greater than 0.2 ng/ml, confirmed by two consecutive measurements, can be defined as biochemical recurrence (BCR). In patients with recurrent disease, accurate evaluation of recurrence location and whole-body tumor burden are essential in patient-specific therapy planning. However, conventional imaging modalities including CT, bone scan, MRI, and more recently choline-PET/CT are all typically negative at low PSA values (5).

To solve this challenging issue, a new molecular imaging technique named prostate specific membrane antigen (PSMA) PET was introduced into clinical practice. This new PET tracer relies on the highly specific expression of PSMA by PCa cells. PSMA is a transmembrane type II glycoprotein, overexpressed in PCa cells, and increased with higher grades, metastasis development, and disease recurrence (6). A series of studies have indicated the priority of this new technique over conventional imaging in the field of primary staging and recurrence location (7). MRI provides much better soft tissue contrast and shows a higher sensitivity in detecting bone metastases in PCa. A combined approach with PSMA PET and mpMRI is capable of acquiring PET and MR data simultaneously or sequentially in a single examination. A potential added value of PSMA PET/MRI can be expected in prostate cancer. Recent studies suggested that PSMA-PET/MRI can provide superior detection efficacy as well as a considerable impact on decision-making (8). Sangwon Han et al. reviewed all studies assessing the impact of PSMA PE/CT and PET/MRI in patients with PCa, and found the proportion of management changes was 54% (9). To our knowledge, the impact of PSMA PET/MRI on the management has not been determined in patients with defined PCa. Moreover, its impact regarding changes in decision-making for patients suspected of PCa has not been assessed. It is important to evaluate the role of PSMA PET/MRI in management changes for wide acceptance of this new technology by referring physicians in clinical practice.

We initially performed simultaneous ^18^F-PSMA-1007 PET/MRI in patients with suspected PCa, primary PCa, and BRPCa patients, and investigated its impact on decision-making. Besides, we explored the potential association between PSA levels and management change.

## Material and Methods

### Patients and Methods

Patients were retrospectively identified and grouped into three subgroups: group 1 comprised patients with suspected PCa (PSA level >4 ng/ml, and/or digital rectal examination abnormality, and/or positive imaging); group 2 included men undergoing primary staging for primary PCa; group 3 comprised patients undergoing imaging for BCR with PSA levels greater than 0.2 ng/ml. Other inclusion criteria: age between 18 to 85 years, ability to understand study procedures, and volunteering to participate in this study. Exclusion criteria were acute prostatitis, the presence of any other concomitant cancers, PSA values less than 0.2 ng/ml, and transurethral resection of prostate (TURP) history. The study was approved by the Ethics Committee of Shanghai Ruijin Hospital (Approved No. 2019-18), and written informed consent was obtained from all patients.

Patient-related clinical information was collected by a urologist with more than 3 years’ experience. Serum PSA levels were recorded closest to the scan. Two records of PSA value for each BRPCa patient within a 12-mo period before the scan were applied to calculate PSA doubling time (PSADT). A questionnaire was adapted from Roach et al. (10) to record management plans before and after PSMA PET/MRI. Management strategies were decided by a multidisciplinary meeting (MDM) consisting of urologists, pathologists, radiologists, and nuclear medicine physicians. All patients underwent a simultaneous ^18^F-FDG PET and mpMRI before PSMA-PET/MRI examination. The initial management strategy was retrospectively decided by MDM discussion according to simultaneous ^18^F-FDG PET and mpMRI results. After PSMA-PET/MRI examination, each pre-planned strategy was modified according to the PSMA-PET/MRI result, and revised managements from MDM discussion were recorded. The impact of PSMA PET/MRI on management was measured as the proportion of patients whose treatment was changed from a previous plan.

### Imaging Protocol and Interpretation

^18^F-PSMA-1007 was produced as described by Cardinale et al. (11). Each patient received an intravenous injection of ^18^F-PSMA-1007 with a median dose of 263 MBq (range 164-353 MBq), then a PET/MRI examination was performed from the vertex to mid-thighs after 60 min of tracer uptake time using an integrated PET/MRI system (Biograph mMR, Siemens Healthcare). All ^18^F-PSMA-1007 PET/MRI images were analyzed independently with dedicated software (Syngovia version VB 10, Siemens Healthcare). In line with published literature, any focal uptake of ^18^F-PSMA-1007 ligand higher than the surrounding background without correspondence to physiologic uptake was considered positive. Two experienced nuclear medicine physicians (M.Z., B.L.) interpreted the ^18^F-PSMA-1007 PET/MRI images, and disagreements were resolved by consensus.

### Management Decision Review

Based on the NCCN guidelines strictly, both the initial management plan and the revised management plan were made by MDM discussion (two urologist, one pathologist, one radiologist, and two nuclear medicine physicians), and all disagreements were resolved by consensus. For patients with suspected PCa, management decisions were categorized as active surveillance (AS) and prostate biopsy. A prostate biopsy was suggested for patients with elevated PSA levels (more than 4 ng/ml), or digital rectal examination abnormality, or positive imaging. For defined PCa patients, management decisions were categorized as active surveillance (AS), surgery (radical prostatectomy with or without pelvic lymph nodes dissection), salvage radiotherapy (sRT), systemic therapy (anti-androgen therapy or chemotherapy), and multimodal therapy (more than one type of the therapies mentioned above). Radical prostatectomy (RP) was a standard therapy for primary localized or locally advanced PCa. Systemic therapy was considered when patients with positive lymph nodes (LNs) out of pelvic and/or distant metastases in patients with primary PCa. For BRPCa patients, when the imaging was negative, AS, sRT, or ADT were selected according to clinical treatment history or doctor’s experience. Systemic therapy or multimodal therapy was considered when imaging was positive. Additionally, simultaneous integrated boost intensity-modulated RT (SIB-IMRT) was considered when imaging was positive in the prostate bed or pelvic LNs. Stereotactic body radiotherapy (SBRT) is also considered as an option in oligometastatic patients.

### Statistical Analysis

All the demographic and clinical data were assessed by descriptive analysis. For continuous variables, medians and interquartile range (IQR) were reported. For categorical variables, counts and percentages were calculated. PSADT was calculated according to the method described by Khan et al. (12). All analysis was assessed using SPSS software (version 22.0.0, IBM Corp., Armonk, NY, USA) and R 3.6.2 framework. Relationships between clinical variables and positive rates or management change accessed by Fisher’s exact test, and two-side p < 0.05 was assumed as statistical significance.

## Results

### Patient Characteristics

**Figure 1:**
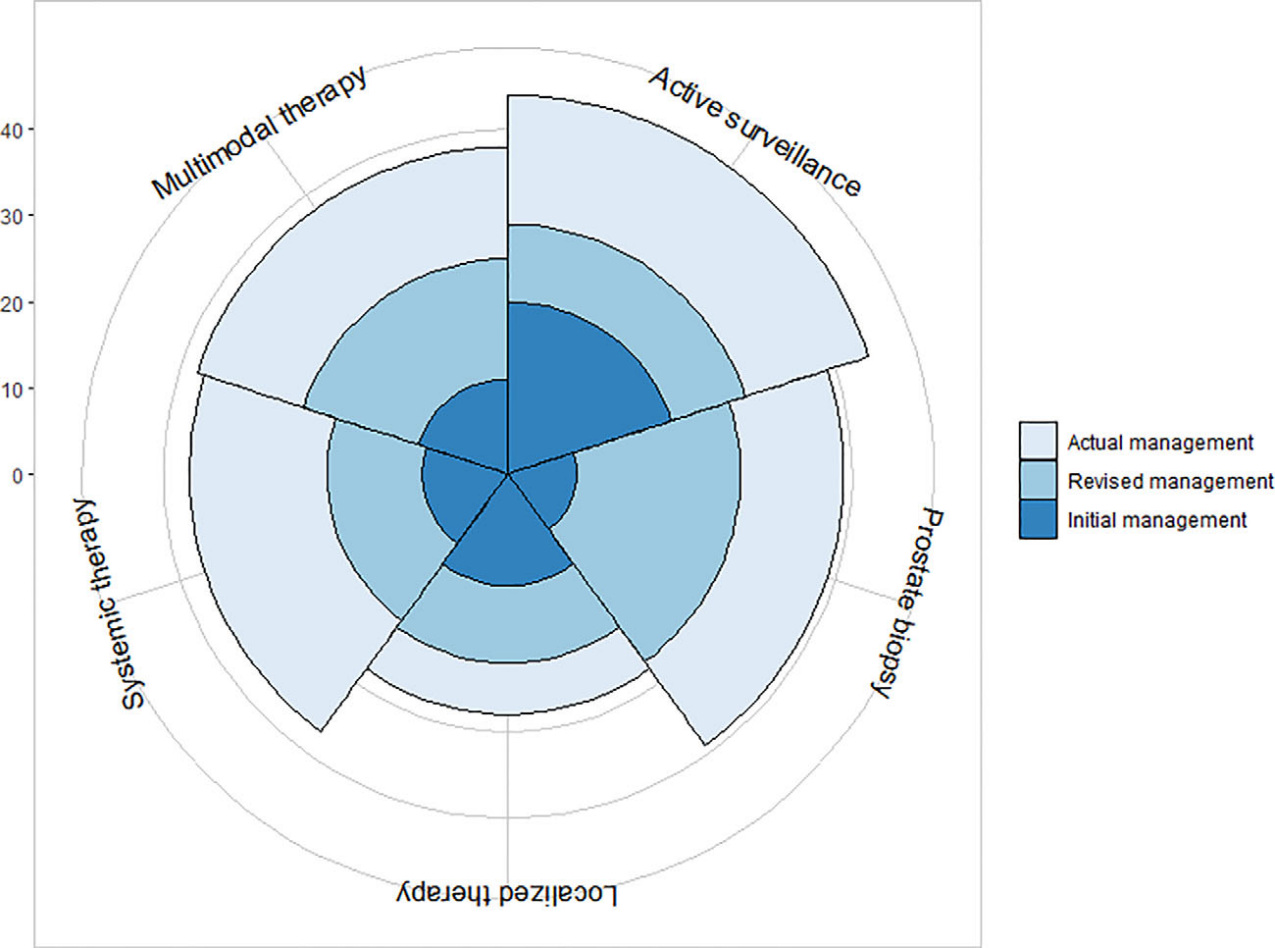
Rose diagram shows the distribution of managements before 18F-PSMA-1007 PET/MRI (initial management), after 18F-PSMA-1007 PET/MRI (revised management), and implemented management (actual management). Management decisions were categorized as active surveillance, prostate biopsy, localized therapy (surgery and salvageable pelvic radiotherapy), systemic therapy (anti-androgen therapy or chemotherapy), and multimodal therapy (more than one therapy type).

From June 2019 to June 2020, 62 consecutive patients who underwent PSMA-PET/MRI were retrospectively identified. The basic information of patients was summarized in **Table 1**. There were 28 patients in group 1 with median age of 63.5 years (IQR 60.5–68.0 years), and median PSA level of 9.8 ng/ml (IQR 6.5–13.1). Fifteen (53.6%) patients in group 1 had received a prostate biopsy in the past. There were 12 patients in group 2 with median age of 68.5 years (IQR 64.5–73.8 years), and median PSA level of 29.9 ng/ml (IQR 7.0–100.7). Four patients in group 2 had distant metastasis. Moreover, five patients in group 2 were receiving ADT at PET/MRI. There are 22 patients in group 3 with median age of 70.5 years (IQR 63.0–75.8 years), and median PSA level of 2.0 ng/ml (IQR 0.94–4.67). In 17 (77.3%) of 22 patients, the initial treatment was curative therapy, and in 5 (22.7%) of 22 patients, the initial treatment was ADT. There were 15 patients in group 3 were receiving ADT at PET/MRI, and 19 patients had ADT history. Management change details and follow-up information were presented in **Table 2**. A rose diagram shows the distribution of managements before PSMA PET/MRI, after PSMA PET/MRI, and implemented management in **Figure 1**. The management changes of each patient were detailed in **Supplementary Figure 1**.

**Table 1:** Basic characteristics of patients.

Clinical variable	Suspected PCa(n = 28)	Primary PCa(n = 12)	BRPCa(n = 22)
Mean age, years, (IQR)	63.5 (60.5–68.0)	68.5 (64.5–75.8)	70.5 (63.0–75.8)
Median PSA, ng/ml, (IQR)	9.8 (6.5–13.1)	29.9 (7.0–100.7)	2.0 (0.9–4.7)
Median PSAdt, months, (IQR)	/	/	2.1 (1.5–5.6)
ISUP group, n (%)	/		
2		5 (41.7)	5 (22.7)
3		1 (8.3)	6 (27.3)
4		1 (8.3)	2 (9.1)
5		4 (33.3)	5 (4.5)
NA		1 (8.3)	4 (18.1)
Tumor stage, n (%)	/		
T2		6 (50.0)	7 (31.8)
T3		1 (8.3)	11 (50.0)
T4		5 (41.7)	3 (13.6)
NA		0	1 (4.5)
Nodal stage, n (%)	/		
N0		7 (58.3)	16 (72.7)
N1		5 (41.7)	5 (22.7)
NA		0	1 (4.5)
Metastasis stage	/		
M0		8 (66.7)	17 (77.3)
M1		4 (33.3)	4 (18.2)
NA		0	1 (4.5)
Previous management, n (%)			
Prostate biopsy	15 (53.6)	12 (100)	22 (100)
Curative therapy	0	0	17 (77.3)
ADT history, n (%)	0	0	17 (77.3)
Ongoing ADT, n (%)	0	4 (33.3)	13 (59.1)

Note. PCa, prostate cancer; BRPCa, biochemical recurrence prostate cancer; PSA, prostate specific antigen; ADT, androgen deprivation therapy; PSADT, prostate specific antigen doubling time.

**Table 2:** Management before and after ^18^F-PSMA-1007 PET/MRI in patients with suspected PCa, primary PCa, and BRPCa.

Management blinded to PSMA	Revised management plan	Implemented management	Follow-up PSA or pathological evolution
**Patients with suspected PCa (n = 28)**			
AS (20)	AS (9)	AS (9)	8↓and 1→
	Prostate biopsy (11)	AS (2) Prostate biopsy (8) RP (1)	1↑and 1NA 4 pos and 4 neg 1 pos
Prostate biopsy (8)	Prostate biopsy (8)	AS (3) Prostate biopsy (4) Systemic therapy (1)	3→ 2 pos and 2 neg 1↓
**Primary PCa patients (n =12)**			
Surgery (8)	Surgery (7)	Surgery (5) Systemic therapy (2)	4↓↓and 1↓ 1↓↓and 1↓
	Multimodal therapy (1)	Systemic therapy (1)	1↓
Systemic therapy (4)	Systemic therapy (4)	AS (1) Systemic therapy (3)	1↓ 3↓
**BRPCa patients (n = 22)**			
sRT (5)	sRT (2)	Systemic therapy (2)	2↓↓
	Multimodal therapy (3)	Multimodal therapy (3)	3↓↓
Systemic therapy (6)	Systemic therapy (6)	Systemic therapy (5) Multimodal therapy (1)	2↑and 2↓and 1→ 1→
Multimodal therapy (11)	Systemic therapy (1)	Systemic therapy (1)	1↓↓
	Multimodal therapy (10) (all with minor change)	Systemic therapy (1) Multimodal therapy (9)	1↑ 5↓↓and 2↓and 2↑

PCa, prostate cancer; BRPCa, biochemical recurrence prostate cancer; AS, Active surveillance; RP, Radical prostatectomy; sRT, salvage radiotherapy.

PSA evolution, ↑ (increased PSA level); ↓ (measurable decreased PSA level); ↓↓(indosable PSA level);→ (stable PSA level); NA (not available).

Pathological evolution : pos (positive pathology); neg (negative pathology).

### Changes in Suspected Prostate Cancer

**Figure 2:**
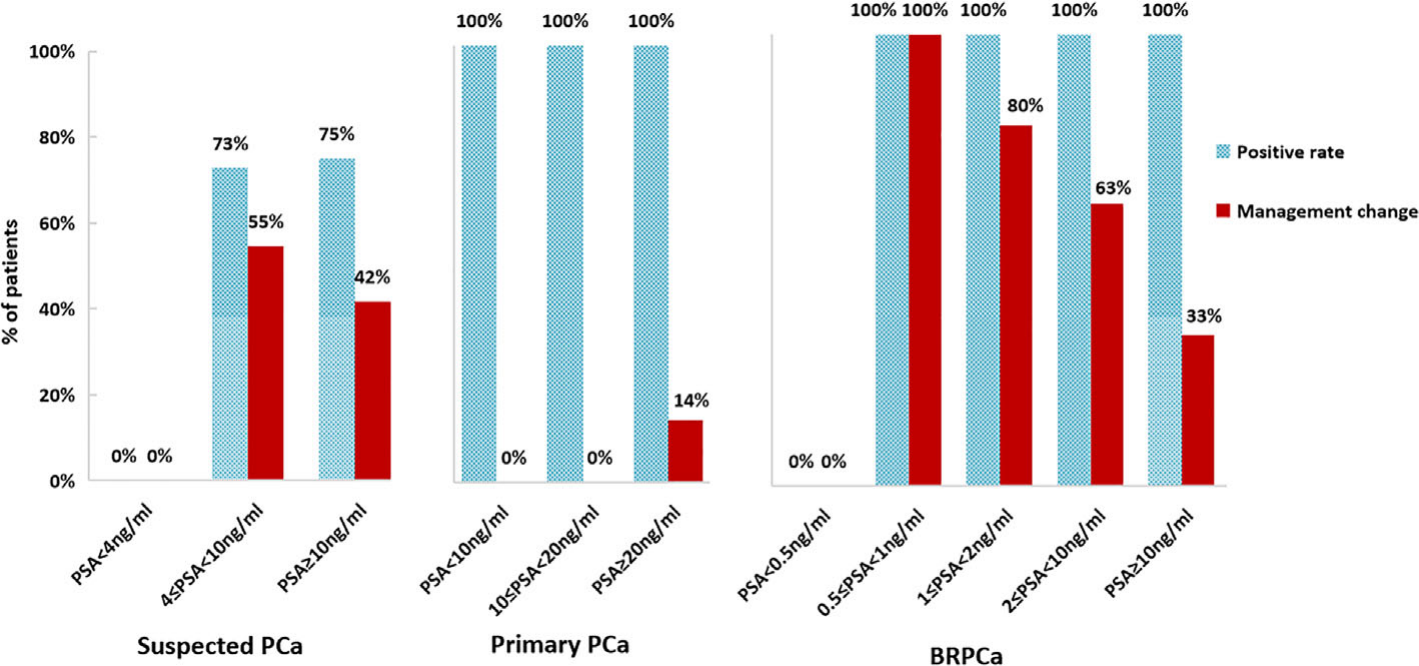
Positive rates and management change proportions at different PSA levels in suspected PCa, primary PCa, and BRPCa.

**Figure 3:**
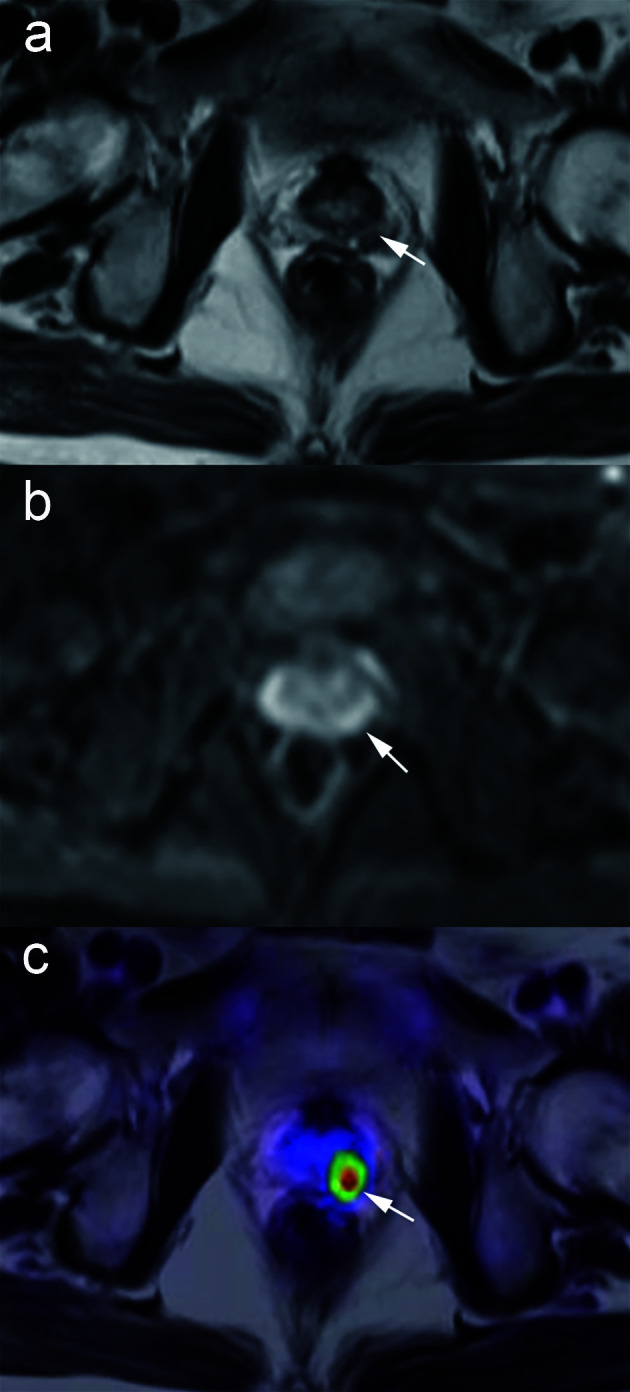
A patient treated with management change in suspected PCa. Images from a 59-year-old male with a PSA level of 7.07 ng/ml. No tumor detection within the prostate is achievable with T2-weighted or DWI sequence alone **(A**, **B)**, but fused PET/MRI demonstrates tumor involvement of the left lobe (**C**, white arrow). The management plan was shifted from active surveillance to biopsy. The subsequent prostate biopsy confirmed Gleason 4 + 3 prostate cancer in the ipsilateral lobe.

^18^F-PSMA-1007 PET/MRI was positive in 17 (60.7%) patients and negative in 11 (39.3%) patients. ^18^F-PSMA-1007 PET/MRI resulted in a change of management in 11 (39.3%) patients. Before PSMA, 8 patients planned to perform prostate biopsy, and 20 patients planned to undergo AS. After ^18^F-PSMA-1007 PET/MRI, we suggested 19 patients perform prostate biopsy, and 9 patients to perform AS. In 27 suspected PCa patients with PSA data before PSMA PET/MRI, the positive rates were 0, 73, and 75% with PSA levels of <4 ng/ml, 4 ≤ PSA < 10 ng/ml, and PSA ≥ 10 ng/ml, respectively. The proportions of management changes were 0, 55, and 42% with PSA <4 ng/ml, 4 ≤ PSA < 10 ng/ml, and PSA ≥ 10 ng/ml, respectively (**Figure 2**). There was a significant association between PSA groups and PSMA positivity (p = 0.027). Higher PSA levels were not associated with decision-making changes (p = 0.865). A patient who shifted treatment was exemplified in **Figure 3**.

Follow-up is available for a median of 5.5 months (range 4–15 months) in 28 suspected patients. Details of management implementation were given in **Figure 1** and **Table 2**. There were 13 patients underwent biopsy (one patient have RP directly), 14 patients insisted on active surveillance, and one patient without pathological evidence underwent ADT directly, and was followed with decreased PSA level. Finally, seven patients were confirmed as PCa, six patients were negative for PCa. For the three positive patients who insisted on active surveillance, one patient had increased PSA (up to 13.8 ng/ml), one patient was lost, and one patient had stable PSA (19.2 ng/ml). For the 11 patients with negative PSMA PET/MRI, all of them selected AS, and no PCa was found till the last follow-up date. The follow-up PSA evolution and pathology evolution were detailed in **Table 2**.

### Changes in Primary Prostate Cancer

^18^F-PSMA-1007 PET/MRI was positive in all primary patients and resulted in a change of management in 1 (8.3%) patient. One patient shifted management from RP with PLND to multimodal therapy because of the detection of oligometastatic lesions. As shown in **Figure 2**, No management changes occurred in patients with PSA less than 20 ng/ml. Only in the category of PSA levels of ≥20 ng/ml, the management of primary PCa was changed (14% of patients).

Follow-up is available for a median of 9 months (range 4–15 months) in 12 primary PCa patients. Details of management implementation and follow-up PSA evolution were given in **Figure 1** and **Table 2**. PSMA PET/MRI identified localized or locally advanced PCa in seven patients, and PCa with distant metastases in five patients. In the seven patients without metastases, five patients underwent RP and the majority was followed with undetectable PSA level (defined as <0.008 ng/ml), one patient with advanced PCa was planned to underwent neoadjuvant complete androgen blockade (CAB) for 3 to 6 months, and one patient with life expectancy <5 years also received CAB therapy. In the five patients with distant metastases, four patients with systemic therapy was followed with decreased PSA level, one patient was treated with Chinese traditional medicine and was followed with decreased PSA level.

### Changes in Biochemical Recurrent Prostate Cancer

**Figure 4:**
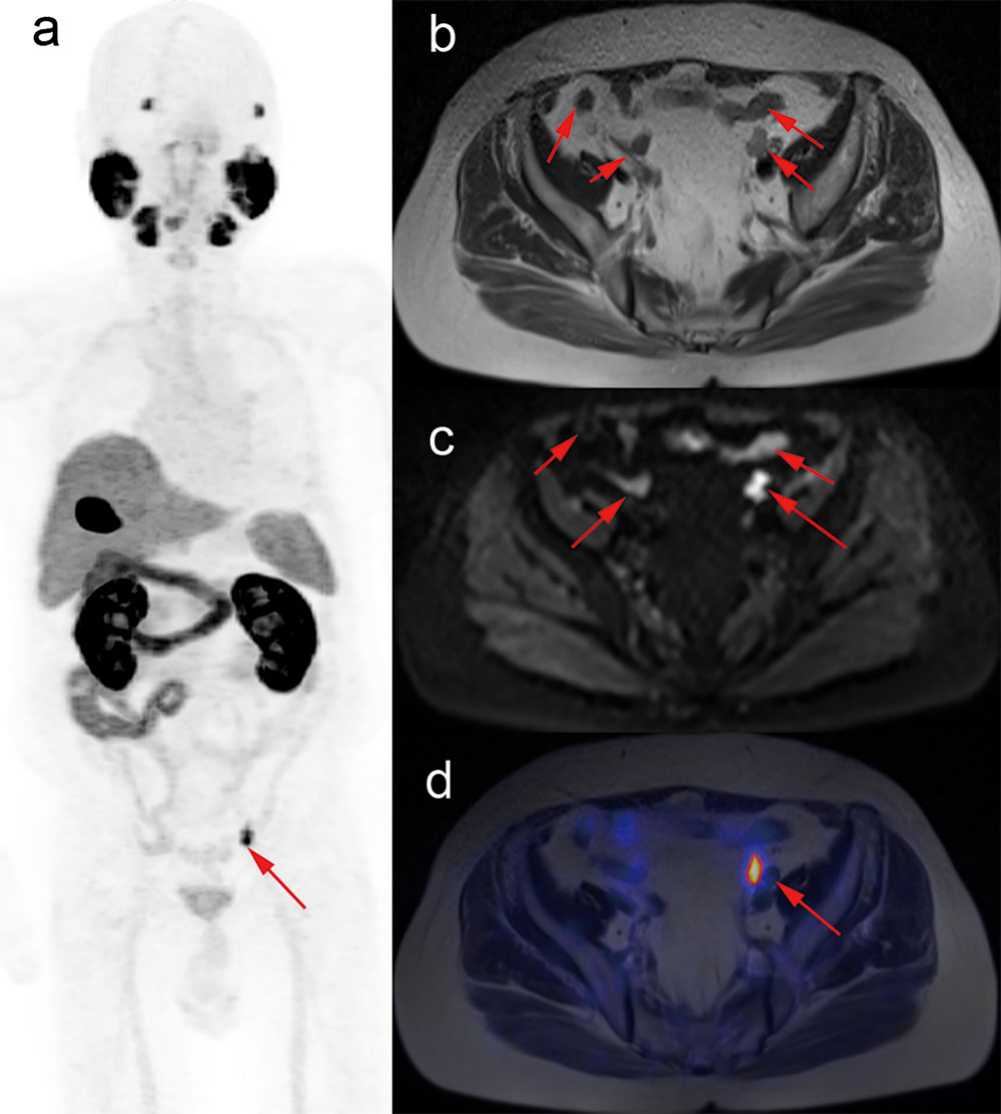
A patient treated with management change in BRPCa. Images from 79-year-old male after radical prostatectomy (June 2018, Gleason score 4 + 4), following with combined androgen blockade therapy, and with PSA values rising to 0.375 ng/ml (August 2019). T2-weighted and DWI images show multiple suspicious nodes in the pelvis **(B, C)**. However, maximum-intensity projection of ^18^F-PSMA-1007 PET and fused PET/MRI images show intense tracer-associated uptake in only two lymph nodes (**A**, **D**, red arrow). Management plan was revised from androgen deprivation therapy combined with external beam radiotherapy to salvage pelvic lymph nodes dissection in combination with external beam radiotherapy.

In the BRPCa group, ^18^F-PSMA-1007 PET/MRI was positive in 20 (90.9%) of 22 patients. Fourteen (63.6%) of 22 patients changed management plans after the examination. In three patients with positive finding beyond pelvis, the initial pelvic radiotherapy was changed into multimodal therapy. One patient in the multimodal group shifted to systemic therapy, and the other 10 patients in multimodal therapy also had minor management changes (the combination of therapy types changed, or the same treatment types with more or less aggressive/extended approach), as exemplified in **Figure 4**. Finally, the number of patients of sRT decreased from 5 to 2, systemic therapy increased from 6 to 7, and multimodal therapy increased from 11 to 13. No patients with PSA values less than 0.5 ng/ml had positive imaging, and all patients with PSA values of ≥0.5 ng/ml had at least one positive lesion. Management change rate ranged from 0 to 100% for the several categories of PSA levels. The highest proportion of management change occurred in patients with 0.5 ≤ PSA < 1 ng/ml (**Figure 2**). Higher PSA levels were significantly associated with positive results (p = 0.004). There was no significant association between PSA and management change (p = 1.000). We also explored the relation between PSADT and positive rates or proportions of management change, though only 16 patients had sufficient information. From patients with PSADT levels of ≤3 months to >3 months, the positive rate decreased from 90.9 to 80.0% and the proportion of management change decreased from 63.6 to 40.0%. PSADT categories were not significantly associated with positive rates (p = 1.000) or proportions of management change (p = 0.596).

Follow-up is available for a median of 9 months (range 4–15 months) in 22 BRPCa patients. Details of management implementation were given in **Figure 1** and **Table 2**. Two patients with negative ^18^F-PSMA-1007 PET/MRI results selected combined androgen blockade (CAB) therapy, as there was ongoing concern about regional recurrent disease due to a continuing rise in PSA, and finally achieved undetectable PSA level. In the 20 patients with positive PSMA-PET/MRI imaging, 7 patients underwent CAB therapy or ADT plus abiraterone therapy, and 13 patients underwent multimodal therapy. The majority of patients with multi-modal treatment received ADT and sRT except one patient who received salvage PLND combined with CAB therapy. The follow-up PSA evolution was detailed in **Table 2**.

### Management Plans Remained After ^18^F-PSMA-1007 Positron Emission Tomography/Magnetic Resonance Imaging

Concerning the patients whose treatment plans were not revised after ^18^F-PSMA-1007 PET/MRI, group 1 included 17/28 patients, group 2 included 11/12 patients, and group 3 included 8/22 patients. In the suspected PCa group, nine patients with negative PSMA results remained AS, and eight patients met the criteria of biopsy and were suggested to perform prostate biopsy. In group 2, surgery was already planned in seven patients with localized PCa, and subsequent PSMA PET/MRI confirmed localized disease. Four patients with multiple metastases were unfit for focal treatment after the initial evaluation and then confirmed by the PSMA PET/MRI. In group 3, there were six patients with multiple metastasis remained systemic therapy, and two patients with both negative conventional imaging and PSMA-PET/MRI remained sRT. The plans that were not altered are presented in detail in **Table 3**.

**Table 3:** Management plans that were not altered after ^18^F-PSMA-1007 PET/MRI.

Patient types	Management plan	Reasons
Suspected PCa (17)	Prostate biopsy (8) AS (9)	Negative PSMA results (9); PSA >4 ng/ml and/or abnormal nodes in prostate and/or positive imaging (8).
Primary PCa (11)	Systemic therapy (4) Surgery (7)	Polymetastasis was seen on conventional imaging, PSMA detected more lesions but without influence on management (4); PSMA confirmed localized PCa (7).
BRPCa (8)	sRT (2) Systemic therapy (6)	BCR patients with negative imaging (2) CRPC patients with polymetastasis (6).

PCa, prostate cancer; BRPCa, biochemical recurrence prostate cancer; CRPC, castration resistant prostate cancer; EBRT, external beam radiotherapy; PLND, pelvic lymph nodes dissection; sRT, salvageable radiotherapy.

## Discussion

Accurate detection of tumor existence, tumor staging as well as the recurrent lesions is crucial for patients before initiation of any kind of management. PET/MRI has emerged as a promising molecular imaging technique being explored in the field of prostate cancer (9). Despite a relatively small sample size, we reports that ^18^F-PSMA-1007 PET/MRI could change the clinical decision‐making in 39.3% of suspected PCa patients, 8.4% of primary PCa patients, and 63.6% of BRPCa patients. Our results indicate that the biggest impact caused by ^18^F-PSMA-1007 PET/MRI on decision-making occurred in the BRPCa group, especially in patients with 0.5 ≤ PSA <1 ng/ml. A review of the literature shows that 27 studies also reported the impact of PSMA-PET on management in patients with primary PCa or BRPCa, but only one looked at primary PCa patients was based on PSMA-PET/MRI (**Supplementary Table 1**). To our knowledge, this is the first study to explore the impact of simultaneous ^18^F-PSMA-1007 PET/MRI on clinical management in suspected PCa, primary PCa, and BRPCa patients. Totally, we conducted a real-life clinical utility of ^18^F-PSMA-1007 PET/MRI in PCa field and it has been shown to be promising and useful tools in the clinical decision making of PCa patients, especially for BRPCa patients.

### Changes in Suspected Prostate Cancer

In recent years, several small-scale reports have successively confirmed the application of PSMA-PET in suspected PCa. Especially, PSMA-PET guided prostate biopsy may be a valuable alternative to improve the detection rate of clinically significant prostate cancer (csPCa). Le-Le Zhang et al. included 60 patients with suspected PCa, 25 patients with positive results underwent PSMA-PET guided target biopsy. Finally, PCa and csPCa were detected in 21/60 (35.0%) and 20/60 (33.3%) patients, respectively (13). Chen Liu et al. investigated 31 suspected PCa patients with prior negative biopsy. All patients underwent PSMA PET-ultrasound fusion image-guided biopsy. Imaging was positive in 18 patients, and csPCa was detected in 12 of 31 patients (38.7%) (14). Lopci et al. prospectively observed 45 patients suspicious for prostate cancer. The cohort comprised men with equivocal mpMRI and at least one negative biopsy. Twenty-five patients (55.5%) with positive results underwent PSMA-PET guided prostate biopsy, and the detection rate of prostate cancer was 44% (15). In our study, 13/17 patients with positive results underwent biopsy (including one patient who underwent RP directly). Finally, seven (53.8%) and six (46.2%) patients were confirmed as PCa and csPCa (Gleason score 7 or greater), respectively. Compared with Lopci et al., we included three patients with positive MRI and only 15 of 23 (53.6%) patients had negative biopsy history, which may partly explain the high positive rates of imaging and pathology. After ^18^F-PSMA-1007 PET/MRI, there was an increase in the use of prostate biopsy and a decrease in the use of AS. Our results indicate that PSMA PET/MRI may improve the detection rate of PCa and avoid unnecessary biopsy.

### Changes in Primary Prostate Cancer

Many published data confirmed the performance of PSMA PET regarding the detection of lymph node and distant metastases in staging before surgery. The treatment modification was due to the high sensitivity of the PSMA-PET for small distant metastatic spread. Kulkarni et al. prospectively investigated 50 patients with high-risk PCa. Of the 50 patients, 12 (24%) had management changed after PSMA PET/CT imaging (16). Hofman et al. designed a randomized phase 3 study, and recruited men with high-risk PCa in Australia, the result provided compelling evidence that PSMA-PET/CT conferred management change in 41/148 (28%) patients (17). In our study, PSMA-PET/MRI changed the clinical strategy in 8.3% of the patients with primary PCa, which was lower than Hofman’s and Kulkarni’s study. On the one hand, the number of the patient was quietly limited in this subgroup. On the other hand, we analyzed all primary PCa patients, rather than focused on high-risk PCa, as high-risk PCa is more likely to develop metastasis. This may underestimate the impact on management change. In our study, only one patient with PSA >20 ng/ml changed management after imaging, no management change happened in patients with PSA <20 ng/ml. However, Kulkarni et al. demonstrated that patients with PSA <20 ng/ml had more frequent management changes than PSA >20 ng/ml, which was contrary to ours. The relationship between PSA and management change in primary PCa is still inconclusive.

### Changes in Biochemical Recurrent Prostate Cancer

Our study found that the biggest impact of management intent was in patients with BRPCa, with a 63.6% intended management change noted. We found that PSMA-PET/MRI detected no site of uptake in patients with PSA levels less than 0.5 ng/ml, whereas published literature described detection rates in the order of 45–60% (18). All patients with PSA levels of more than 0.5 ng/ml had positive images, suggested the great performance of ^18^F-PSMA-1007 PET/MRI. A meta-analysis showed the pooled detection rate of ^18^F-labeled PSMA PET/CT was 49% for PSA <0.5 ng/ml and 86% for PSA ≥0.5 ng/ml (19). There are two possible explanations for the different positive rates between meta-analysis and our reports. On the one hand, our patient number is too limited. On the other hand, the detection rates of PSMA PET in BRPCa patients influenced by many heterogeneous factors, such as received ADT before PSMA-PET, types of tracer (^68^Ga or ^18^F labeled), scan model (PET/MRI or PET/CT), or have undergone either RP and RT history. The impact of PSMA PET on the management in BRPCa patients has been widely evaluated. Overall management impact has been reported in the range from 51 to 76% (9, 20). In the present study, management change occurred in 63.6% BRPCa patients, which was comparable with other published studies. Moreover, the concomitant administration of ADT in patients, PET positivity, PSA levels, and PSADT had recently been reported as the most common heterogeneous source of management change. Our result suggested that management changes occurred mostly in patients with 0.5 ≤ PSA <1 ng/ml. For patients with PSA >1 ng/ml, there is a decreased trend of the proportion of management change in BRPCa patients. One possible explanation for the trend may be the advantage of PSMA-PET/MRI over conventional imaging is not obvious at a high recurrent PSA level, and the proportion of management change decreased. This finding was consistent with the EAU guideline, which suggested PSMA PET in BRPCa patients with lower recurrent PSA levels.

Previous studies on this new technology have mostly been based on ^68^Ga-PSMA-11 PET/CT, while studies focusing on ^18^F-PSMA-1007 PET/MRI were less numerous. Compared with ^68^Ga-labeled radiotracers, ^18^F-PSMA-1007 has a longer half-life, is easily available, and has significant hepatobiliary clearance (21). Therefore, ^18^F-PSMA-1007 PET/MRI may have advantages in detecting local recurrence and easily popularize in clinical practice. Our study evaluated changes between the intended management plan and the revised plan after PSMA PET/MRI, then indicated the clinical value of PSMA PET/MRI. However, a prior study suggested that the implanted management was quite different from the revised treatment plan (22). Studies evaluated the impact of this new technology on actual management is also necessary. Additionally, a cost-effectiveness analysis has to be addressed in a dedicated evaluation before clinical recommendation. Moreover, whether the treatment decision based on PSMA-PET/MRI is beneficial for longer or better survival have yet to be concluded. A multicenter phase III trial (SPPORT trial) in patients with BCR showed freedom-from-progression rate increased from 71.7% in patients who received prostate bed radiation alone to 89.1% in patients who received prostate bed radiation, pelvic lymph node radiation and short-term ADT (23). Such changes in practice could mean that PSMA-PET may add survival benefit when extra-pelvic oligometastatic lesions are detected which may benefit from targeted radiation (24). Further studies are warranted to elucidate whether the change of management will directly translate into survival benefit.

Some limitations of the present study should be noted. Firstly, the patient number is lower than previous studies, with a median patient number of 117 patients (range 15–431) per study, which affects the confidence of our results. However, we report a 41.9% of management change, which is comparable to previous studies **(** **Supplementary Figures 2**-**3** **)**. This limitation can be explained by that only preliminary results from our institution are presented, and will disappear once our future larger prospective study is completed (ChiCTR2000036425). For the same reason, to date, no long-term follow‐up is available. Secondly, the lack of histological validation is a common limitation in imaging studies. Only a part of patients in suspected and primary PCa groups have pathological confirmation. We were unable to report confirmed pathological data in the BRPCa patients of PSMA-positive lesions due to ethical reasons. Certainly, our study was also limited by the retrospective nature. Finally, our patient cohort was heterogeneous. For one thing, we included patients of suspected PCa, primary PCa, and BRPCa. For another, types of initial treatments in BRPCa patients were also different (including curative and palliative therapy). Nonetheless, this showed a real-life situation that physicians always preferred to apply new imaging technology into different types of patients, and then the best appropriate indications were identified.

## Conclusion

From our preliminary experience, PSMA-PET/MRI altered intended decision-making in 39.3% of patients with suspected PCa, 8.3% of patients with primarily diagnosed PCa, and 63.6% patients with BRPCa respectively. The biggest impact of management intent was in patients with BRPCa, especially in patients with 0.5 ≤ PSA <1 ng/ml. This result indicated that PSMA-PET/MRI could be a valued tool for defining lesions in the PCa field and making a personalized clinical decision. However, further larger studies are needed to confirm our pilot findings.

## Data Availability Statement

The original contributions presented in the study are included in the article/**Supplementary Material**; further inquiries can be directed to the corresponding authors.

## Ethics Statement

The studies involving human participants were reviewed and approved by the Ethics Committee of Shanghai Ruijin Hospital (Approved No. 2019-18). The patients/participants provided their written informed consent to participate in this study. Written informed consent was obtained from the individual(s) for the publication of any potentially identifiable images or data included in this article.

## Author Contributions

DX had full access to all the data in the study and takes responsibility for the integrity of the data and the accuracy of the data analysis. DX and AL conceptualized and designed the study. MZ and HH acquired the data. HH and LC analyzed and interpreted the data. AL and LC drafted the manuscript. BL and DX critically revised the manuscript for important intellectual content. WL and XR peformed the statistical analysis. DX and BL obtained the funding. CZ provided administrative, technical, or material support. AL and XR supervised the study. All authors contributed to the article and approved the submitted version.

## Funding

This study was funded by the National Natural Science Foundation of China (No. 81972405) and the Shanghai Committee of Science and Technology, China (No. 18411960100).

## Conflict of Interest

The authors declare that the research was conducted in the absence of any commercial or financial relationships that could be construed as a potential conflict of interest.

## Supplementary Material

Supplementary Figure 1 Details of managements before ^18^F-PSMA-1007 PET/MRI, after ^18^F-PSMA-1007 PET/MRI, and implemented managements based on per patient. AS, active surveillance; RP, radical prostatectomy; sRT, salvage radiotherapy.

Supplementary Figure 2 Summarize related literatures, regarding the positive rate and management impact of PSMA-PET in PCa patients. Our results were in line with previous studies.

Supplementary Figure 3 Point diagram describes the positive rate and management impact of PSMA-PET in PCa patients at per study level.

## References

[1] Siegel RL Miller KD Jemal A. Cancer statistics, 2019. CA: Cancer J Clin (2019) 69:7–34. 10.3322/caac.21551 30620402

[2] Chen W Zheng R Baade PD Zhang S Zeng H Bray F. Cancer statistics in China, 2015. CA: Cancer J Clin (2016) 66:115–32. 10.3322/caac.21338 26808342

[3] Kasivisvanathan V Rannikko AS Borghi M Panebianco V Mynderse LA Vaarala MH. MRI-Targeted or Standard Biopsy for Prostate-Cancer Diagnosis. N Engl J Med (2018) 378:1767–77. 10.1056/NEJMoa1801993 PMC9084630 29552975

[4] Stabile A Giganti F Rosenkrantz AB Taneja SS Villeirs G Gill S. Multiparametric MRI for prostate cancer diagnosis: current status and future directions. Nat Rev Urol (2020) 17:41–61. 10.1038/s41585-019-0212-4 31316185

[5] Zacho HD Nielsen JB Afshar-Oromieh A Haberkorn U deSouza N De Paepe K. Prospective comparison of (68)Ga-PSMA PET/CT, (18)F-sodium fluoride PET/CT and diffusion weighted-MRI at for the detection of bone metastases in biochemically recurrent prostate cancer. Eur J Nucl Med Mol Imaging (2018) 45:1884–97. 10.1007/s00259-018-4058-4 29876619

[6] Perner S Hofer MD Kim R Shah RB Li H Moller P. Prostate-specific membrane antigen expression as a predictor of prostate cancer progression. Hum Pathol (2007) 38:696–701. 10.1016/j.humpath.2006.11.012 17320151

[7] Pernthaler B Kulnik R Gstettner C Salamon S Aigner RM Kvaternik H. A Prospective Head-to-Head Comparison of 18F-Fluciclovine With 68Ga-PSMA-11 in Biochemical Recurrence of Prostate Cancer in PET/CT. Clin Nucl Med (2019) 44:e566–e73. 10.1097/rlu.0000000000002703 31283605

[8] Grubmuller B Baltzer P Hartenbach S D'Andrea D Helbich TH Haug AR. PSMA Ligand PET/MRI for Primary Prostate Cancer: Staging Performance and Clinical Impact. Clin Cancer Res (2018) 24:6300–7. 10.1158/1078-0432.CCR-18-0768 30139879

[9] Han S Woo S Kim YJ Suh CH. Impact of 68Ga-PSMA PET on the Management of Patients with Prostate Cancer: A Systematic Review and Meta-analysis. Eur Urol (2018) 74:179–90. 10.1016/j.eururo.2018.03.030 29678358

[10] Roach PJ Francis R Emmett L Hsiao E Kneebone A Hruby G. The impact of68Ga-PSMA PET/CT on management intent in prostate cancer: Results of an australian prospective multicenter study. J Nucl Med (2018) 59:82–8. 10.2967/jnumed.117.197160 28646014

[11] Giesel FL Hadaschik B Cardinale J Radtke J Vinsensia M Lehnert W. F-18 labelled PSMA-1007: biodistribution, radiation dosimetry and histopathological validation of tumor lesions in prostate cancer patients. Eur J Nucl Med Mol Imaging (2017) 44:678–88. 10.1007/s00259-016-3573-4 PMC5323462 27889802

[12] Khan MA Carter HB Epstein JI Miller MC Landis P Walsh PW. Can prostate specific antigen derivatives and pathological parameters predict significant change in expectant management criteria for prostate cancer? J Urol (2003) 170:2274–8. 10.1097/01.ju.0000097124.21878.6b 14634395

[13] Zhang LL Li WC Xu Z Jiang N Zang SM Xu LW. (68)Ga-PSMA PET/CT targeted biopsy for the diagnosis of clinically significant prostate cancer compared with transrectal ultrasound guided biopsy: a prospective randomized single-centre study. Eur J Nucl Med Mol Imaging (2020). 10.1007/s00259-020-04863-2 PMC7835307 32734457

[14] Liu C Liu T Zhang Z Zhang N Du P Yang Y. (68)Ga-PSMA PET/CT Combined with PET/Ultrasound-Guided Prostate Biopsy Can Diagnose Clinically Significant Prostate Cancer in Men with Previous Negative Biopsy Results. J Nucl Med (2020) 61:1314–9. 10.2967/jnumed.119.235333 PMC7456174 32034111

[15] Lopci E Saita A Lazzeri M Lughezzani G Colombo P Buffi NM. (68)Ga-PSMA Positron Emission Tomography/Computerized Tomography for Primary Diagnosis of Prostate Cancer in Men with Contraindications to or Negative Multiparametric Magnetic Resonance Imaging: A Prospective Observational Study. J Urol (2018) 200:95–103. 10.1016/j.juro.2018.01.079 29409824

[16] Kulkarni M Hughes S Mallia A Gibson V Young J Aggarwal A. The management impact of (68)gallium-tris(hydroxypyridinone) prostate-specific membrane antigen ((68)Ga-THP-PSMA) PET-CT imaging for high-risk and biochemically recurrent prostate cancer. Eur J Nucl Med Mol Imaging (2020) 47:674–86. 10.1007/s00259-019-04643-7 PMC7005085 31872280

[17] Hofman MS Lawrentschuk N Francis RJ Tang C Vela I Thomas P. Prostate-specific membrane antigen PET-CT in patients with high-risk prostate cancer before curative-intent surgery or radiotherapy (proPSMA): a prospective, randomised, multicentre study. Lancet (2020) 395:1208–16. 10.1016/s0140-6736(20)30314-7 32209449

[18] Wang R Shen G Yang R Ma X Tian R. (68)Ga-PSMA PET/MRI for the diagnosis of primary and biochemically recurrent prostate cancer: A meta-analysis. Eur J Radiol (2020) 130:109131. 10.1016/j.ejrad.2020.109131 32622250

[19] Treglia G Annunziata S Pizzuto DA Giovanella L Prior JO. Detection Rate of (18)F-Labeled PSMA PET/CT in Biochemical Recurrent Prostate Cancer: A Systematic Review and a Meta-Analysis. Cancers (2019) 11(5):710. 10.3390/cancers11050710 PMC6562935 31126071

[20] Hoffmann MA Wieler HJ Baues C Kuntz NJ Richardsen I Schreckenberger M. The Impact of 68Ga-PSMA PET/CT and PET/MRI on the Management of Prostate Cancer. Urology (2019) 130:1–12. 10.1016/j.urology.2019.04.004 30986486

[21] Freitag MT Kesch C Cardinale J Flechsig P Floca R Eiber M. Simultaneous whole-body (18)F-PSMA-1007-PET/MRI with integrated high-resolution multiparametric imaging of the prostatic fossa for comprehensive oncological staging of patients with prostate cancer: a pilot study. Eur J Nucl Med Mol Imaging (2018) 45:340–7. 10.1007/s00259-017-3854-6 29038888

[22] Calais J Fendler WP Eiber M Gartmann J Chu FI Nickols NG. Impact of (68)Ga-PSMA-11 PET/CT on the Management of Prostate Cancer Patients with Biochemical Recurrence. J Nucl Med (2018) 59:434–41. 10.2967/jnumed.117.202945 PMC5868499 29242398

[23] Pollack A Karrison TG Balogh AG Low D Bruner DW Wefel JS. Short Term Androgen Deprivation Therapy Without or With Pelvic Lymph Node Treatment Added to Prostate Bed Only Salvage Radiotherapy: The NRG Oncology/RTOG 0534 SPPORT Trial. Int J Radiat Oncol Biol Phys (2018) 102:1605–. 10.1016/j.ijrobp.2018.08.052

[24] Jadvar H. Oligometastatic Prostate Cancer: Molecular Imaging and Clinical Management Implications in the Era of Precision Oncology. J Nucl Med (2018) 59:1338–9. 10.2967/jnumed.118.213470 PMC6126444 30030344

[25] Bianchi L Schiavina R Borghesi M Ceci F Angiolini A Chessa F. How does (68) Ga-prostate-specific membrane antigen positron emission tomography/computed tomography impact the management of patients with prostate cancer recurrence after surgery? Int J Urol (2019) 26:804–11. 10.1111/iju.14012 31083784

[26] Rousseau E Wilson D Lacroix-Poisson F Krauze A Chi K Gleave M. A Prospective Study on (18)F-DCFPyL PSMA PET/CT Imaging in Biochemical Recurrence of Prostate Cancer. J Nucl Med (2019) 60:1587–93. 10.2967/jnumed.119.226381 PMC6836862 30979820

[27] Schmidt-Hegemann NS Eze C Li M Rogowski P Schaefer C Stief C. Impact of (68)Ga-PSMA PET/CT on the Radiotherapeutic Approach to Prostate Cancer in Comparison to CT: A Retrospective Analysis. J Nucl Med (2019) 60:963–70. 10.2967/jnumed.118.220855 PMC6604695 30552203

[28] Müller J Ferraro DA Muehlematter UJ Garcia Schüler HI Kedzia S Eberli D. Clinical impact of (68)Ga-PSMA-11 PET on patient management and outcome, including all patients referred for an increase in PSA level during the first year after its clinical introduction. (2019) 46:889–900. 10.1007/s00259-018-4203-0 30488099

[29] Mattiolli AB Santos A Vicente A Queiroz M Bastos D Herchenhorn D. Impact of 68GA-PSMA PET / CT on treatment of patients with recurrent / metastatic high risk prostate cancer - a multicenter study. Int Braz J Urol (2018) 44:892–9. 10.1590/s1677-5538.ibju.2017.0632 PMC6237542 30088720

[30] Farolfi A Ceci F. (68)Ga-PSMA-11 PET/CT in prostate cancer patients with biochemical recurrence after radical prostatectomy and PSA <0.5 ng/ml. Efficacy and impact on treatment strategy. Eur J Nucl Med Mol Imaging (2019) 46:11–9. 10.1007/s00259-018-4066-4 29905907

[31] Roach PJ Francis R Emmett L Hsiao E Kneebone A Hruby G. The Impact of (68)Ga-PSMA PET/CT on Management Intent in Prostate Cancer: Results of an Australian Prospective Multicenter Study. J Nucl Med (2018) 59:82–8. 10.2967/jnumed.117.197160 28646014

[32] Hope TA Aggarwal R Chee B Tao D Greene KL Cooperberg MR. Impact of (68)Ga-PSMA-11 PET on Management in Patients with Biochemically Recurrent Prostate Cancer. J Nucl Med (2017) 58:1956–61. 10.2967/jnumed.117.192476 28522741

[33] Habl G Sauter K Schiller K Dewes S Maurer T Eiber M. (68) Ga-PSMA-PET for radiation treatment planning in prostate cancer recurrences after surgery: Individualized medicine or new standard in salvage treatment. Prostate (2017) 77:920–7. 10.1002/pros.23347 28317152

[34] Albisinni S Artigas C Aoun F Biaou I Grosman J Gil T. Clinical impact of (68) Ga-prostate-specific membrane antigen (PSMA) positron emission tomography/computed tomography (PET/CT) in patients with prostate cancer with rising prostate-specific antigen after treatment with curative intent: preliminary analysis of a multidisciplinary approach. BJU Int (2017) 120:197–203. 10.1111/bju.13739 27981732

[35] van Leeuwen PJ Stricker P Hruby G Kneebone A Ting F. (68) Ga-PSMA has a high detection rate of prostate cancer recurrence outside the prostatic fossa in patients being considered for salvage radiation treatment. BJU Int (2016) 117:732–9. 10.1111/bju.13397 26683282

[36] Fendler WP Ferdinandus J Czernin J Eiber M Flavell RR Behr SC. Impact of (68)Ga-PSMA-11 PET on the Management of Recurrent Prostate Cancer in a Prospective Single-Arm Clinical Trial. J Nucl Med (2020) 61:1793–9. 10.2967/jnumed.120.242180 PMC9364898 32358094

[37] Grubmüller B Baltzer P D’Andrea D Korn S Haug AR Hacker M. (68)Ga-PSMA 11 ligand PET imaging in patients with biochemical recurrence after radical prostatectomy - diagnostic performance and impact on therapeutic decision-making. Eur J Nucl Med Mol Imaging (2018) 45:235–42. 10.1007/s00259-017-3858-2 PMC5745568 29075832

[38] Shakespeare TP. Effect of prostate-specific membrane antigen positron emission tomography on the decision-making of radiation oncologists. Radiat Oncol (2015) 10:233. 10.1186/s13014-015-0548-8 26582424 PMC4652371

[39] Bluemel C Linke F Herrmann K Simunovic I Eiber M Kestler C. Impact of (68)Ga-PSMA PET/CT on salvage radiotherapy planning in patients with prostate cancer and persisting PSA values or biochemical relapse after prostatectomy. EJNMMI Res (2016) 6:78. 10.1186/s13550-016-0233-4 27785766 PMC5081978

[40] Sterzing F Kratochwil C Fiedler H. 68)Ga-PSMA-11 PET/CT: a new technique with high potential for the radiotherapeutic management of prostate cancer patients. (68)Ga-PSMA-11 PET/CT: a new technique with high potential for the radiotherapeutic management of prostate cancer patients. Eur J Nucl Med Mol Imaging (2016) 43:34–41. 10.1007/s00259-015-3188-1 26404016 PMC4771815

[41] Rousseau C Le Thiec M Ferrer L Rusu D Rauscher A Maucherat B. Preliminary results of a (68) Ga-PSMA PET/CT prospective study in prostate cancer patients with occult recurrence: Diagnostic performance and impact on therapeutic decision-making. Prostate (2019) 79:1514–22. 10.1002/pros.23869 31421657

[42] Dewes S Schiller K Sauter K Eiber M Maurer T Schwaiger M. Integration of (68)Ga-PSMA-PET imaging in planning of primary definitive radiotherapy in prostate cancer: a retrospective study. Radiat Oncol (2016) 11:73. 10.1186/s13014-016-0646-2 27229485 PMC4882861

[43] Gauthé M Belissant O Girard A Zhang Yin J Ohnona J Cottereau AS. [PET/CT and biochemical recurrence of prostate adenocarcinoma: Added value of (68)Ga-PSMA-11 when (18)F-fluorocholine is non-contributive]. Progres en Urol J l’Assoc Fr d’urol la Soc Fr d’urol (2017) 27:474–81. 10.1016/j.purol.2017.04.004 28576423

[44] Henkenberens C Derlin T Bengel FM Ross TL Wester HJ Hueper K. Patterns of relapse as determined by (68)Ga-PSMA ligand PET/CT after radical prostatectomy : Importance for tailoring and individualizing treatment. Strahlentherapie und Onkol Organ der Dtsch Rontgengesellschaft [et al] (2018) 194:303–10. 10.1007/s00066-017-1231-9 29134231

[45] Song H Harrison C Duan H Guja K Hatami N Franc BL. Prospective Evaluation of (18)F-DCFPyL PET/CT in Biochemically Recurrent Prostate Cancer in an Academic Center: A Focus on Disease Localization and Changes in Management. J Nucl Med (2020) 61:546–51. 10.2967/jnumed.119.231654 31628216

[46] Zacho HD Nielsen JB Dettmann K Haberkorn U Langkilde NC Jensen JB. 68Ga-PSMA PET/CT in Patients With Biochemical Recurrence of Prostate Cancer: A Prospective, 2-Center Study. Clin Nucl Med (2018) 43:579–85. 10.1097/rlu.0000000000002169 29916917

[47] Mena E Lindenberg ML Shih JH Adler S Harmon S Bergvall E. Clinical impact of PSMA-based (18)F-DCFBC PET/CT imaging in patients with biochemically recurrent prostate cancer after primary local therapy. Eur J Nucl Med Mol Imaging (2018) 45:4–11. 10.1007/s00259-017-3818-x 28894899 PMC7983162

