# Supplementary figures and images for: Clinical Utility of 18F-PSMA-1007 Positron Emission Tomography/Magnetic Resonance Imaging in Prostate Cancer: A Single-Center Experience
Source: Front Oncol. 2021 Feb 11;10:612701. doi: 10.3389/fonc.2020.612701 (PMC7928386; doi:10.3389/fonc.2020.612701)

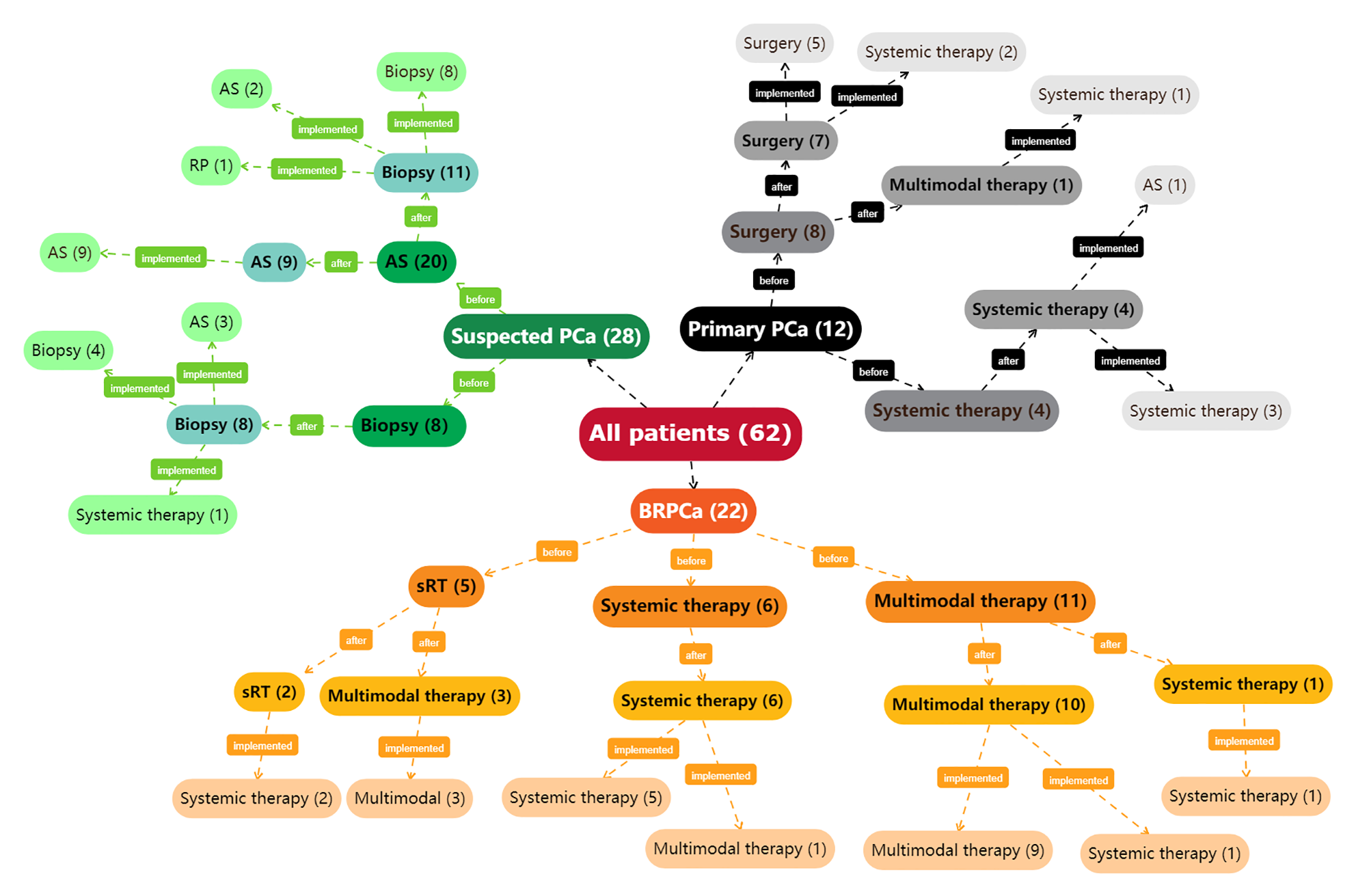

Supplement: Supplementary Figure 1 — Details of managements before 18F-PSMA-1007 PET/MRI, after 18F-PSMA-1007 PET/MRI, and implemented managements based on per patient. AS, active surveillance; RP, radical prostatectomy; sRT, salvage radiotherapy. [file Image_1.tif]

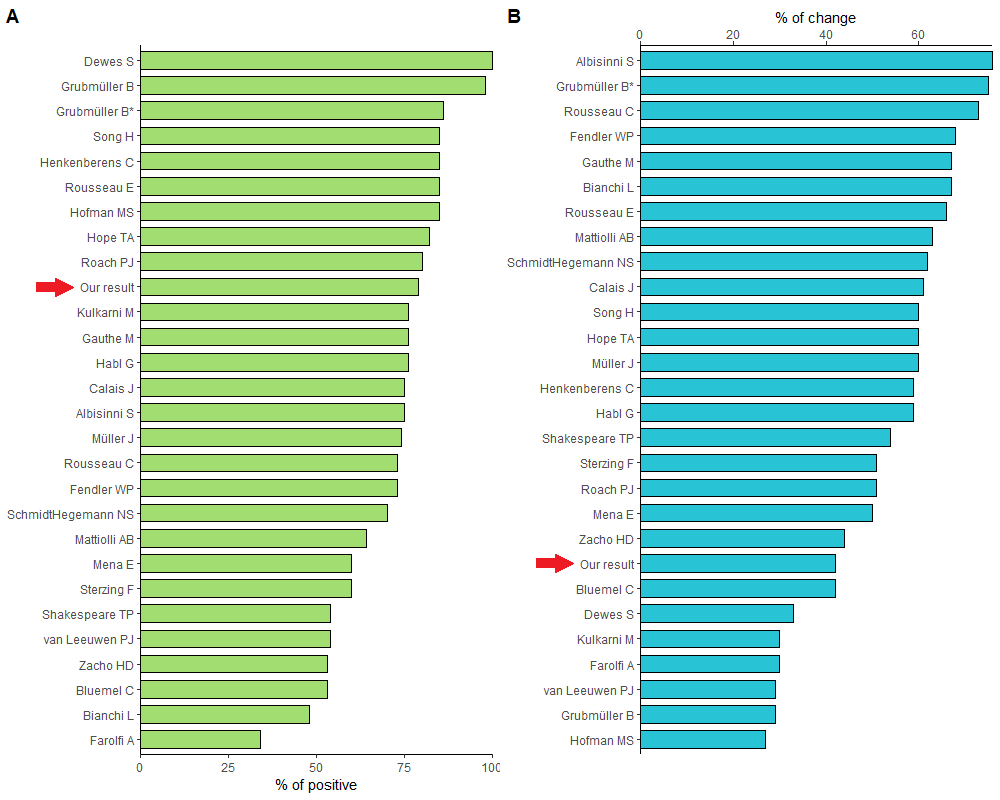

Supplement: Supplementary Figure 2 — Summarize related literatures, regarding the positive rate and management impact of PSMA-PET in PCa patients. Our results were in line with previous studies. [file Image_2.tif]

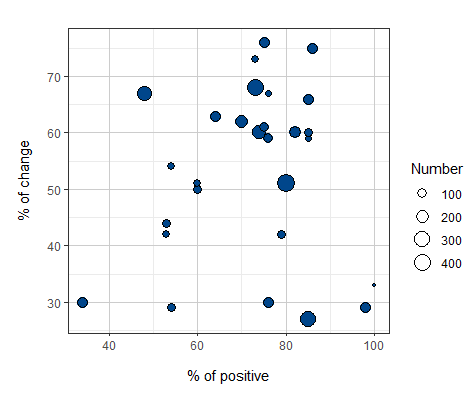

Supplement: Supplementary Figure 3 — Point diagram describes the positive rate and management impact of PSMA-PET in PCa patients at per study level. [file Image_3.tiff]
