# Supplementary material for: Clinical Utility of 18F-PSMA-1007 Positron Emission Tomography/Magnetic Resonance Imaging in Prostate Cancer: A Single-Center Experience
Source: Front Oncol. 2021 Feb 11;10:612701. doi: 10.3389/fonc.2020.612701 (PMC7928386; doi:10.3389/fonc.2020.612701)
Supplement: Supplementary file 4 [file Table_1.docx]

**Supplement table 1. Literatures review. The positive rate and management impact of PSMA-PET in PCa patients.**

| **First author** | **Clinical setting** | **No.**  **patients** | **Scan** | **%PSMA PET +** | **%management Changes** | **Follow-up clinical impact** |
| --- | --- | --- | --- | --- | --- | --- |
| Hofman MS^[17]^ | High-risk PCa | 302 | ^68^Ga-PSMA PET/CT | 85.0 | 27.0 | NA |
| Bianchi L^[^[^25^](#_ENREF_26)^]^ | BRPCa | 276 | ^68^Ga-PSMA PET/CT | 47.5 | 66.6 | NA |
| Rousseau E^[^[^2^](#_ENREF_27)^6]^ | BRPCa | 130 | ^18^F-DCFPyL PET/CT | 84.6 | 65.5 | NA |
| SchmidtHegemann NS^[^[^2^](#_ENREF_28)^7]^ | Primary PCa and BRPca | 172 | ^68^Ga-PSMA PET/CT | 70.0 | 62.0 | NA |
| Müller J^[^[^2^](#_ENREF_29)^8]^ | BRPCa | 223 | ^68^Ga-PSMA PET | 74.0 | 60.0 | complete response (PSA<0.2 ng/ml) after 6 months in 45% patients |
| Grubmüller B^[^[^8^](#_ENREF_30)^]^ | Primary PCa | 122 | ^68^Ga-PSMA PET/MRI | 97.5 | 28.7 | NA |
| Mattiolli AB ^[^[^29^](#_ENREF_31)^]^ | High-risk PCa and BRPCa | 125 | ^68^Ga-PSMA PET/CT | 64.0 | 63.4 | NA |
| Farolfi A^[^[^3^](#_ENREF_32)^0]^ | BRPCa with PSA <0.5ng/ml | 119 | ^68^Ga-PSMA PET/CT | 34.4 | 30.2  (radiotherapy) | NA |
| Roach PJ^[^[^3^](#_ENREF_33)^1]^ | Primary PCa and BRPCa | 431 | ^68^Ga-PSMA PET/CT | 80 | 51 | No change in 40%; worse prognosis in 36%;improved prognosis in 9% |
| Hope TA^[^[^3^](#_ENREF_34)^2]^ | BRPCa | 150 | ^68^Ga-PSMA PET | 81.7 | 59.6 | NA |
| Habl G^[^[^3^](#_ENREF_35)^3]^ | BRPCa | 100 | ^68^Ga-PSMA PET | 76.0 | 59.0  (radiotherapy ) | NA |
| Albisinni S^[^[^3^](#_ENREF_36)^4]^ | BRPCa | 131 | ^68^Ga-PSMA PET/CT | 75.0 | 76.0 | NA |
| van Leeuwen PJ^[^[^3^](#_ENREF_37)^5]^ | BRPCa | 70 | ^68^Ga-PSMA PET/CT | 54.0 | 28.6 | NA |
| Fendler WP^[^[^3^](#_ENREF_38)^6]^ | BRPCa | 382 | ^68^Ga-PSMA PET | 73.0 | 68.0 | NA |
| Grubmüller B*^[^[^3^](#_ENREF_39)^7]^ | BRPCa | 117 | ^68^Ga-PSMA PET | 85.5 | 74.6  (radiotherapy) | 3 month PSA 0.17 (IQR 0.04-0.41)  6 month PSA (IQR 0.03-0.73)  12 month PSA (IQR 0.04-1.04) |
| Shakespeare TP^[38]^ | PCa and BRPCa | 54 | ^68^Ga-PSMA PET | 53.7 | 53.7  (radiotherapy) | NA |
| Bluemel C^[39]^ | BRPCa | 45 | ^68^Ga-PSMA PET/CT | 53.3 | 42.2  (radiotherapy) | ↓PSA in 95.3% pts |
| Sterzing F^[^[^4^](#_ENREF_42)^0]^ | PCa and BRPCa | 57 | ^68^Ga-PSMA PET/CT | 73.5 | 60.5  (radiotherapy) | NA |
| Calais J^[^[^22^](#_ENREF_22)^]^ | BRPCa | 101 | ^68^Ga-PSMA PET/CT | 75.0 | 61.0 | NA |
| Rousseau C^[^[^4^](#_ENREF_43)^1]^ | BRPCa | 52 | ^68^Ga-PSMA PET/CT | 73.1 | 73.1 | Undetectable PSA after treatment in 19.2% pts; ↓PSA>60% in 34.6% pts |
| Dewes S^[^[^4^](#_ENREF_44)^2]^ | PCa | 15 | ^68^Ga-PSMA PET | 100 | 33.3 | NA |
| Gauthe M^[^[^4^](#_ENREF_45)^3]^ | BRPCa | 33 | ^68^Ga-PSMA PET/CT | 76.0 | 67.0 | NA |
| Henkenberens C^[^[^4^](#_ENREF_46)^4]^ | BRPCa | 39 | ^68^Ga-PSMA PET/CT | 84.6 | 59.0 | NA |
| Song H^[^[^4^](#_ENREF_47)^5]^ | BRPCa | 72 | ^18^F-DCFPyL PET/CT | 84.7 | 60 | NA |
| Zacho HD^[^[^4^](#_ENREF_48)^6]^ | BRPCa | 70 | ^68^Ga-PSMA PET/CT | 52.9 | 43.5 | NA |
| Kulkarni M^[^[^16^](#_ENREF_16)^]^ | High-risk PCa and BRPCa | 118 | ^68^Ga-PSMA PET/CT | 76.3 | 29.7 | Hhigh-risk PCa: the majority of patients has an undetectable PSA.  BRPCa: in 61 pts. with positive imaging, follow-up has not been discordant with PSMA-PET/CT  In 7 pts. with negative imaging, two patients had increased PSA, and others were on watchful waiting |
| Mena E^[^[^4^](#_ENREF_49)^7]^ | BRPCa | 68 | ^18^F–DCFBC PET/CT | 60.3 | 50.0 | NA |

PCa: prostate cancer; BRPCa: biochemical recurrence prostate cancer; AS: Active surveillance; PSA: Prostate specific antigen; NA: Not available

Note:* two studies from Grubmüller B et al. were summarized in the table.* means the study described the results of BRPCa patients.
